# Hospital Disaster Preparedness and Response to a Pediatric Mass Casualty Incident in Central India: A Retrospective Quality Improvement Analysis

**DOI:** 10.7759/cureus.109124

**Published:** 2026-05-18

**Authors:** Nitin Marathe, Ajay Ambalakkatte, Mahendra Chauhan, Shilpa PN, Nilesh Jagne, Soumya Ghoshal

**Affiliations:** 1 Hospital Administration, All India Institute of Medical Sciences, Nagpur, Nagpur, IND; 2 Department of Trauma and Emergency, All India Institute of Medical Sciences, Nagpur, Nagpur, IND; 3 Department of General Surgery, All India Institute of Medical Sciences, Nagpur, Nagpur, IND

**Keywords:** disaster preparedness, hospital preparedness, mass casualty incident, pediatric trauma, surge capacity, triage

## Abstract

Background

Mass casualty incidents (MCIs) place extreme demands on health systems, and pediatric victims introduce additional physiological and psychosocial challenges. Although disaster preparedness principles are well described, reports of coordinated pediatric MCI responses from India remain limited. On 26 November 2024, a school bus accident near Nagpur led to the simultaneous arrival of 49 casualties at a tertiary trauma center. This study uses retrospective operational data to evaluate hospital surge capacity, triage, imaging workflow, and clinical outcomes and interprets these findings in relation to selected published reports on MCI response.

Methods

A retrospective observational analysis was conducted using hospital operational logs, emergency department triage records, and electronic medical data for all 49 patients. Variables included notification time, arrival pattern, triage categories, resource utilization, imaging volumes, operative interventions, and clinical outcomes. Categorical data are presented as frequencies and percentages. Findings were interpreted descriptively in relation to selected published MCI reports; no formal statistical benchmark comparison was performed.

Results

The hospital received the alert at 10:15 AM and immediately activated its MCI protocol. Forty-nine casualties (46 students, two volunteers, and one teacher) arrived in rapid succession. A 50-bed surge ward was established, and multidisciplinary teams were mobilized. Baseline imaging evaluation was performed for 48 patients, excluding one patient who was brought dead; 26 patients required computed tomography. One emergency laparotomy for bladder rupture and two operative reductions with internal fixation were performed. Minor procedures included 22 wound dressings, seven sutures, three splint applications, and one foreign body removal (33 minor procedures in total). Within 24 hours, 42 patients (85.7%) were discharged, including one patient discharged against medical advice; six patients (12.2%) required hospital admission, including one pediatric intensive care unit admission following emergency bladder rupture repair, and one patient (2.0%) was brought dead. No in-hospital deaths occurred.

Conclusion

Early activation of a structured disaster plan, rapid surge expansion, coordinated triage with pediatric adaptations, prioritized imaging, and multidisciplinary collaboration were observed during this pediatric MCI response. The high same-day discharge rate and absence of in-hospital mortality among patients arriving alive suggest that institutional preparedness and surge strategies may support MCI response, although causal attribution is limited by the descriptive design and lack of severity-adjusted comparison.

## Introduction

Mass casualty incidents (MCIs) are events in which the number or acuity of patients overwhelms immediately available healthcare resources [1]. They produce sudden surges in patient volume that strain infrastructure, staffing, and supply chains; therefore, hospital surge capacity preparedness encompasses not only physical assets but also organizational processes, communication pathways, and leadership structures [2].

Children are particularly vulnerable during disasters because of anatomical, physiological, and developmental differences that affect injury patterns and treatment requirements [3]. Primary triage systems such as START (Simple Triage and Rapid Treatment) and Care Flight categorize patients by physiological parameters; comparative analyses of multiple‑casualty triage algorithms have reported sensitivities of 82-85% and specificities of 86-96%, with the motor component of the Glasgow Coma Scale and systolic blood pressure being highly predictive [4]. A scoping review concluded, however, that no single primary triage algorithm reliably identifies critically injured children, and only one secondary algorithm demonstrated superior performance [5].

Field triage guidelines incorporate physiologic, anatomic, and mechanism‑of‑injury criteria, emphasizing emergency medical services judgment [6]. Nevertheless, prospective validation of those guidelines showed an overall sensitivity of 66.2% and specificity of 87.8% for identifying patients with an injury severity score ≥16, with sensitivity falling to 51.8% among adults aged ≥55 years and rising to 87.4% among children, highlighting the need for clinical oversight and secondary triage [7].

Surge capacity can be conceptualized through the “4S” framework-staff, supplies, structures, and systems-and its strengthening requires sustained preparedness investments [8]. The “walking wounded” phenomenon, where most victims sustain minor injuries and self‑present, can overwhelm emergency services unless victims are rapidly segregated [9]. Indian tertiary‑care series reports approximately 66% emergency department discharges, 19% admissions, and 5% intensive care utilization, offering useful benchmarks [10].

Imaging logistics profoundly influence patient throughput during MCIs. Standard whole-body computed tomography (CT) protocols can process approximately six patients per hour per scanner [11]. Accelerated triage CT pathways reduce total scan time to 8.9 minutes per patient [12]. Reconstruction and picture archiving and communication system (PACS) transfer may delay image availability by approximately 15 minutes [11]. Guidelines recommend that CT be reserved for hemodynamically stable patients and that plain radiographs suffice for the walking wounded, ensuring that imaging supports rather than impedes triage [13].

Pediatric disaster readiness requires more than appropriate equipment; it demands tailored care, pediatric‑specific training, and mental health support [3]. Low‑fidelity pediatric MCI simulations using the JumpSTART algorithm and two‑dimensional patient models have been shown to improve learners’ triage accuracy and confidence, illustrating the value of simulation‑based preparedness [14].

The aim of this study was to evaluate the operational response and hospital disaster preparedness of AIIMS Nagpur during the pediatric MCI on 26 November 2024. The specific objectives were to describe the triage distribution of casualties, assess surge capacity activation and hospital resource mobilization, analyze diagnostic imaging utilization, evaluate procedures and clinical interventions performed, assess patient outcomes including discharge, admission, and intensive care requirement, and identify operational lessons for strengthening institutional disaster preparedness.

This article was previously presented as a research paper at the National Capacity-Building Programme for Disaster-Resilient Hospitals, organized by the Department of Hospital Administration, AIIMS New Delhi, under the aegis of the Disaster Management Cell, Ministry of Health and Family Welfare, Government of India, as part of the national initiative on Health Sector Disaster Preparedness and Response, held at the SET Facility, AIIMS New Delhi, on 16 January 2026.

## Materials and methods

This retrospective observational study used operational data from an MCI that occurred on 26 November 2024 in Nagpur, Maharashtra, India. A school bus carrying students, volunteers, and a teacher overturned following a road traffic collision. All casualties were transported to All India Institute of Medical Sciences (AIIMS), Nagpur, a tertiary-care hospital with a pre-existing MCI protocol.

Data were extracted for all casualties included in the incident response (n=49) from the hospital’s MCI records and incident reports, emergency department triage records, electronic medical records, and radiology records. All patients who arrived at the emergency department as part of the declared MCI were included, irrespective of age, injury severity, triage category, admission status, or final disposition. No patient from the incident was excluded. Extracted data were cross-checked across available operational, emergency department, electronic medical, and radiology records to minimize inconsistencies.

Collected variables included notification time, arrival pattern, patient demographics (students, volunteers, teacher), triage category using START and pediatric-adapted JumpSTART principles where applicable, imaging utilization, surgical and minor procedures, surge bed activation, injury patterns, admission status, intensive care requirement, discharge disposition, and mortality outcomes. Initial triage was performed at the emergency receiving area. Ambulatory patients were initially segregated as walking wounded, while patients requiring urgent assessment, resuscitation, imaging, operative intervention, or admission were directed to appropriate emergency, radiology, surgical, or inpatient care pathways. Triage status was reassessed during clinical evaluation and disposition planning.

For this analysis, surge bed activation was defined as mobilization of additional emergency or inpatient capacity following MCI notification. Imaging utilization was defined as any radiological investigation performed during initial assessment, including extended focused assessment with sonography in trauma (e-FAST), X-ray, CT, or other imaging modalities. Operative intervention refers to emergency surgery performed during the incident response. Admission included ward or intensive care admission for ongoing monitoring or treatment. Same-day discharge was defined as discharge from the emergency department or observation area within 24 hours of arrival. Mortality outcomes were categorized as brought dead/dead on arrival or in-hospital mortality among patients arriving alive.

Categorical variables are summarized as frequencies and percentages. No inferential statistical testing was performed because the study was a single-incident descriptive analysis. Findings were interpreted descriptively in relation to selected published MCI reports; no formal statistical benchmark comparison was performed. Formal injury severity scores, time to imaging, time to surgery, and emergency department length of stay were not consistently available in the retrospective operational records and were therefore not analyzed.

The study used anonymized operational quality-improvement data. Institutional ethics committee approval was obtained with waiver of informed consent.

The findings of this analysis were submitted to the Quality and Patient Safety Committee of AIIMS Nagpur as part of a structured post-incident review. Operational gaps and lessons identified through this analysis were used to inform updates to the institutional MCI protocol and strengthen staff preparedness training. This study therefore constitutes a retrospective quality-improvement analysis embedded within the hospital’s existing quality assurance framework.

## Results

Timeline and surge response

The hospital received notification of the incident at 10:15 AM, and the MCI protocol was activated immediately. A total of 49 casualties, 46 students, two volunteers, and one teacher, arrived in rapid succession, with the first patients reaching the emergency department at approximately 10:40 AM. A 50-bed surge ward was established adjacent to the emergency department. Multidisciplinary teams comprising trauma surgeons, orthopedic surgeons, pediatricians, anesthesiologists, radiologists, nurses, and administrative personnel were rapidly mobilized. Key operational responses are summarized in Table 1.

**Table 1:** Key Response Metrics and Actions (26 November 2024) *Ward admissions and ICU admissions are reported separately. One student with blunt abdominal trauma required emergency laparotomy and pediatric ICU admission following bladder rupture repair. START, Simple Triage and Rapid Treatment; JumpSTART, pediatric adaptation of START

Metric/Action	Details
Incident notification time	~10:15 AM – Alert received from local police and ambulance services
MCI protocol activation	Immediate activation at notification (code alert)
Total casualties received	49 patients (46 students, two volunteers, one teacher)
First patient arrival	~10:40 AM (multiple ambulances arriving simultaneously)
Triage system	Active at ED entrance; patients color‑coded by severity (START/JumpSTART)
Surge beds activated	50 additional beds (temporary emergency ward)
Additional staff mobilized	Multidisciplinary teams from the ED, surgery, orthopedics, pediatrics, radiology, anesthesia, nursing, housekeeping, and administration
Supplies & equipment	Emergency medications, intravenous fluids, dressings, splints, analgesics, and advanced airway equipment
Blood bank status	High alert – units prepared; no transfusions required
Imaging performed	Baseline imaging was performed for 48 patients, excluding one patient brought dead; 26 patients underwent CT scans using a simplified triage-driven imaging protocol
Critical surgeries	One emergency laparotomy for bladder rupture following blunt abdominal trauma
*ICU admissions	1 (2.0%): student to pediatric ICU post bladder rupture repair surgery
*Ward admissions	5 (10.2%): one orthopedic fracture, two blunt chest injuries, one minor head injury, one teacher with polytrauma
Discharged within 24 hours	42 (85.7%) Includes 39 students, two volunteers, and one student discharged against medical advice after family refused admission
Died (Mortality)	1 (2.0%): declared brought dead on arrival
Help desk for families	Established immediately; provided regular updates and emotional support
Media/communications	Hospital administration coordinated with local authorities; press releases and social media updates

Triage and patient flow

Triage using the START and JumpSTART algorithms categorized the majority of patients as low priority (Table 2). Two patients (4.1%) were triaged as red, two (4.1%) as yellow, 44 (89.8%) as green, and one patient (2.0%) was categorized as black and declared brought dead on arrival. A dedicated fast-track area for ambulatory patients helped prevent congestion in the main resuscitation zone.

**Table 2:** Triage Categories (START/JumpSTART) START, Simple Triage and Rapid Treatment; JumpStart, pediatric adaptation of START

Triage Category	Number	Percentage
Red	2	4.1%
Yellow	2	4.1%
Green	44	89.8%
Black	1	2.0%

Imaging utilization

Baseline imaging evaluation was performed for 48 patients, excluding one patient who was brought dead. In total, 26 patients required CT scans. The imaging modalities employed are detailed in Table 3. Extended focused assessment with sonography in trauma (e-FAST) was performed in 42 patients (87.5%), non-contrast CT head in 26 (54.2%), non-contrast CT cervical spine in 19 (39.6%), contrast-enhanced CT torso in two (4.2%), chest radiography in 42 (87.5%), pelvic radiography in 35 (72.9%), and extremity radiography in 16 (33.3%). The imaging workflow followed a simplified triage-driven protocol that prioritized hemodynamically stable patients for CT and used plain radiographs for walking wounded patients, thereby preventing radiology bottlenecks.

**Table 3:** Imaging Studies Utilized *Percentages were calculated among 48 patients who underwent imaging evaluation, excluding one patient brought dead. Some patients required multiple imaging modalities; therefore, percentages do not total 100%. e-FAST, extended focused assessment with sonography in trauma; NCCT, non-contrast computed tomography; CECT, contrast-enhanced computed tomography.

Type of Imaging	Number	Percentage*
e-FAST	42	87.5%
NCCT Head	26	54.2%
NCCT C-spine	19	39.6%
CECT Torso	2	4.2%
Chest X-ray	42	87.5%
Pelvis X-ray	35	72.9%
Extremity X-ray	16	33.3%

Interventions and procedures

Surgical and minor procedures are summarized in Table 4. One emergency laparotomy was performed for bladder rupture, and two operative reductions with internal fixation were carried out for fractures. Additionally, 22 wound dressings, seven suturing procedures, three splint applications, and one foreign body removal were completed, yielding a total of 33 minor procedures performed.

**Table 4:** Procedures Performed ^Some patients underwent more than one procedure. Minor procedures totalled 33. Major procedures included one laparotomy and two operative reductions with internal fixation, performed in one student and one teacher with polytrauma.

Type of Procedure	Number
Suturing	7
Foreign body removal	1
Splinting	3
Wound dressing	22
Operative reduction and internal fixation^^^	2
Laparotomy^^^	1

Injury patterns

Injury characteristics at disposition are shown in Table 5. Soft tissue injuries predominated, affecting 36 patients (73.5%). Five patients (10.2%) sustained bony fractures, while five (10.2%) had no detectable injuries. Blunt abdominal injuries were observed in two patients (4.0%), blunt chest injuries in three patients (6.1%), and traumatic brain injury in one patient (2.0%).

**Table 5:** Injury Characteristics at Disposition ^+^Percentages were calculated using the total study population (n=49). Some patients sustained more than one type of injury; therefore, totals may exceed 100%.

Type of Injury	Number	Percentage^+^
Soft tissue injuries	36	73.5%
Bony injury (fractures)	5	10.2%
No injuries	5	10.2%
Blunt abdominal injury	2	4.0%
Blunt chest injury	3	6.1%
Traumatic brain injury	1	2.0%

Clinical outcomes

Table 6 presents the patient outcomes by the end of the incident day. Forty-two patients (85.7%), including 39 students, two volunteers, and one student discharged against medical advice after family refusal of admission, were discharged within 24 hours following observation and minor treatment. Six patients (12.2%) required ongoing admission, including one student with an orthopedic fracture, one student with a minor head injury, one student with blunt abdominal trauma requiring emergency laparotomy and pediatric intensive care admission, two students with blunt chest trauma, and one teacher with polytrauma. All admitted patients were stabilized and discharged within one week. One student (2.0%) was declared dead on arrival, representing the sole fatality. No in-hospital deaths occurred among patients who arrived alive.

**Table 6:** Patient Outcomes Summary by the End of the Incident Day (26 November 2024)

Outcome Category	Number (%)	Details
Discharged within 24 hours	42 (85.7%)	Includes 39 students, two volunteers, and one student discharged against medical advice after the family refused admission by 6 PM
Admitted for ongoing care	6 (12.2%)	Five students and one teacher hospitalized (one orthopedic fracture, one minor head injury, one student with blunt abdominal trauma and two students with blunt chest trauma, one adult- teacher with polytrauma)
Fatality	1 (2.0%)	One student brought in with fatal injuries, declared dead on arrival

## Discussion

Key findings

This incident describes how a tertiary-care institution implemented a structured response to a pediatric MCI. Early activation of the disaster plan, rapid surge bed expansion, coordinated triage, and prioritized imaging were observed alongside a high same-day discharge rate of 85.7% and absence of in-hospital mortality among patients arriving alive. The sole fatality was a child who had died before arrival. These findings should be interpreted cautiously because this was a descriptive single-center analysis without a comparator or severity-adjusted assessment. Nevertheless, the observed response processes are consistent with disaster medicine literature, suggesting that structured institutional preparedness may support trauma response during periods of resource strain [1]. They are also aligned with reports highlighting the role of simulation-based preparedness training in strengthening MCI response systems [14].

Comparison with published MCIs

The discharge rate in the present incident exceeded the 66% emergency department discharge rate reported from an Indian tertiary-care trauma center [10]. The admission rate of 12.2%, including a single intensive care unit stay, suggests that most casualties had moderate injuries, while structured triage enabled timely identification of patients requiring ongoing care. The predominance of ambulatory casualties in our incident is consistent with mass casualty triage models that classify patients with minor injuries as the “walking wounded” and recommend their early segregation into a separate ambulatory triage area [9]. However, triage during MCIs remains dynamic; patients initially categorized as ambulatory may require reassessment, up-triage, or transfer if their condition deteriorates [9]. Experiences from mass casualty responses further show that early information regarding expected casualty severity may be unreliable, with hospitals sometimes receiving patients beyond the initially anticipated “walking wounded” category, reinforcing the need for flexible response plans, effective communication, and repeated secondary assessment [15].

Pediatric considerations

Children’s unique physiological traits demand pediatric‑specific preparedness [3]. JumpSTART, the pediatric adaptation of START, exists alongside other tools, though no primary algorithm reliably detects all critically injured children [5]. In the present incident, the combination of START and JumpSTART with senior clinician oversight likely optimized resource allocation. Simulation‑based training enhances pediatric readiness: low‑fidelity, in‑situ pediatric MCI exercises improve triage accuracy and provider confidence, and such drills should be integrated into routine hospital disaster preparedness [14]. Simulation‑based emergency department training also strengthens communication and leadership during MCIs, further supporting its inclusion in mandatory curricula [16]. Recent revisions of pediatric Tactical Emergency Casualty Care guidelines offer consensus recommendations for high‑threat incidents, acknowledging persisting evidence gaps [17]. A review of hospital‑based triage tools emphasizes the importance of secondary triage to reduce under‑triage and recommends consistent use of validated instruments [18].

Surge capacity analysis

Surge capacity rests on the four pillars of staff, supplies, structures, and systems [8]. The rapid establishment of a 50‑bed ward and mobilization of multidisciplinary teams reflected effective coordination across departments. Systematic reviews underline that surge preparedness is not merely a resource stockpile but an ongoing process of activation protocol refinement and communication network testing [2]. The walking wounded, if not immediately identified and fast‑tracked, can exhaust resources intended for critically injured patients; our segregated fast‑track zone helped preserve critical care capacity [9].

Imaging throughput

Radiological workflow can become a rate-limiting step during MCIs. Standard whole-body CT protocols accommodate approximately six patients per hour per scanner [11]. Accelerated triage CT protocols can substantially shorten acquisition times [12], although image reconstruction and PACS transfer may further delay image availability. Selective CT utilization, particularly among hemodynamically stable patients, is recommended to avoid radiology bottlenecks [13]. In this incident, the mechanism of injury, including vehicle rollover and one pre-hospital fatality, necessitated CT imaging in a substantial proportion of patients; however, the simplified triage-driven imaging workflow prevented radiology services from becoming a choke point. Recent evidence suggests that point-of-care ultrasound may reduce reliance on radiography in selected fracture assessments and improve imaging efficiency in emergency settings, an approach that could be explored in future MCI protocols [19].

Mortality benchmarking and preventability

Hemorrhage remains the leading cause of preventable trauma death, and ensuring blood bank readiness is essential even when no transfusions are needed [20]. The absence of in‑hospital mortality in this series, despite one pre‑hospital death, suggests that the triage and resuscitation systems functioned effectively. Prospective validation of field triage guidelines continues to reveal under‑triage concerns, highlighting the indispensable role of clinical judgment [7].

System‑level implications

Hospitals must institutionalize disaster preparedness through surge activation checklists, predefined imaging pathways, dedicated communication cells, and regular simulation‑based training. Field triage guidelines emphasize emergency medical services judgment [6]. However, their limited sensitivity mandates secondary triage and continuous performance monitoring [7]. Simulation-based emergency training may strengthen communication, coordination, and leadership during MCIs [16]. Low‑fidelity simulations provide cost‑effective methods for teaching pediatric triage algorithms and equipment such as the Broselow tape [14]. Specialized pediatric Tactical Emergency Casualty Care guidance may inform preparedness for high-threat, resource-constrained, and mass casualty scenarios; however, persistent evidence gaps highlight the need for continued research and context-specific adaptation [17].

Limitations

This study is a single-center descriptive analysis of a single incident involving a cohort of 49 casualties, which limits generalizability and statistical interpretation. Injury severity scores were not consistently recorded, precluding severity-adjusted comparisons. Although injury patterns were available and reported, formal injury severity scoring was not performed. Time-based performance indicators, including time to imaging, time to surgery, and emergency department length of stay, were not consistently available in the retrospective operational records and were therefore not analyzed. Long-term functional and psychological outcomes were not assessed. Data were extracted retrospectively from MCI incident reports and clinical records, which may contain minor inaccuracies. Formal evaluation of triage accuracy was not performed. The lack of a control group prevents causal attribution of outcomes solely to the MCI protocol. Although the hospital response reflected prior preparedness and coordinated activation of the MCI protocol, the predominance of low-acuity and minor injuries should be considered when interpreting the high same-day discharge rate and absence of in-hospital mortality. The quality improvement component of this study was limited to a single retrospective improvement cycle-identification of operational gaps followed by protocol and training updates through the Quality and Patient Safety Committee. Prospective measurement of the impact of these improvements on future MCI responses was beyond the scope of this analysis, and iterative validation will require data from subsequent incidents or simulation exercises.

## Conclusions

Timely activation of a mass casualty plan, surge capacity expansion, structured triage with pediatric adaptations, prioritized imaging pathways, and coordinated multidisciplinary care were observed during this pediatric MCI response in central India. The high same-day discharge rate and absence of in-hospital mortality among patients arriving alive suggest that institutional preparedness and structured surge processes may support MCI response, although causal attribution is limited by the single-center descriptive design, absence of a comparator, and lack of severity-adjusted analysis. Hospitals should consider embedding disaster protocols into routine operations, strengthening simulation-based training, and incorporating pediatric-specific strategies into preparedness planning.
